# Successful Oral Antimalarial Therapy in A 14-Year-Old Child with Blackwater Fever: A Case in a Rural Area of Asmat Regency of Papua, Indonesia: Successful Oral Antimalarial in Blackwater Fever

**DOI:** 10.1016/j.ijregi.2022.03.021

**Published:** 2022-03-31

**Authors:** Sitanaja Raymond, Abed Ricky Hernando Sitompul

**Affiliations:** 1 Regional Public Hospital of Agats, Asmat Regency, Papua, Indonesia; 2 Department of Child Health, Regional Public Hospital of Agats, Asmat Regency, Papua, Indonesia

**Keywords:** Malaria, Plasmodium falciparum, Blackwater fever, Oral antimalarial

## Abstract

**Background:**

Blackwater fever (BWF) is one of the severe forms of malaria manifested by hemoglobinuria that causes dark-colored urine, fever, anemia, jaundice and acute kidney injury. BWF is most commonly associated with *Plasmodium falciparum* infection and its treatment. Parenteral antimalarial therapy is recommended as the treatment of choice for BWF. Here we present the first case of successful oral antimalarial therapy in BWF to the best of our knowledge.

**Case Presentation:**

A 14-year-old boy was hospitalized with BWF as the primary diagnosis based on the presence of fever, jaundice and “coca-cola”-colored urine, along with laboratory results which showed *Plasmodium falciparum* infection, anemia, and impaired kidney function. Uncomplicated malaria manifestations had been appearing for seven days before admission, but the syndrome of BWF developed several hours following the first dose of dihydroartemisinin-piperaquine (DHP). Treatment with a 3-day course of DHP was continued because parenteral antimalarials were unavailable at that time. Remarkable improvements were seen following the second and third doses of DHP along with adequate supportive medical care.

**Conclusion:**

The unavailability of parenteral antimalarials makes oral antimalarials a possible alternative treatment for BWF. In addition, close monitoring and supportive medical care are critical in the treatment of BWF.

## Highlights

• Artemisinin derivatives are considered to be a risk factor for blackwater fever (BWF).
• Oral antimalarial drugs may be a possible alternative treatment for BWF.
• Close monitoring and supportive medical care are critical in treating BWF.

## Background

Blackwater fever (BWF) is one of the severe forms of malaria manifested by hemoglobinuria that causes dark-colored urine, fever, anemia, jaundice and acute kidney injury (AKI) (Bodi et al., 2013; Lon et al., 2014). BWF is most commonly associated with *Plasmodium falciparum* infection (Barber et al., 2016; Huggan et al., 2018). The exact mechanisms of BWF remain unclear, but several studies have linked it to the use of amino-alcohol antimalarials (especially quinine), glucose-6-phosphate-dehydrogenase (G6PD) deficiency, and, less frequently, artemisinin derivatives (Bodi et al., 2013; Lon et al., 2014; Mahamadou et al., 2019; Olupot et al., 2017). BWF is dangerous and life-threatening, requiring immediate treatment with a parenteral antimalarial (WHO, 2021). To the best of our knowledge, this is the first case report of successful oral antimalarial therapy in BWF.

## Case Presentation

A 14-year-old boy presented to the emergency room (ER) at Regional Public Hospital of Agats in Asmat Regency, Papua, with the chief complaint of weakness that had been worsening for 30 minutes before admission. The patient had reported experiencing fever, chills, headache, myalgia, arthralgia, and diarrhea for one week before admission and had not taken any medication. The patient's mother brought the patient to the clinic that morning. The patient was diagnosed with *Plasmodium falciparum* malaria with a parasite density of 4+ (more than 10 parasites per high power field (HPF)), then the patient was given dihydroartemisinin-piperaquine (DHP) for three days and a single dose of primaquine, along with other supportive drugs. Those were taken once before he was finally taken to the ER. The patient also had a history of *Plasmodium falciparum* malaria, which was treated with the same DHP and primaquine regimens last year.

The patient looked agitated and had a Glasgow Coma Scale (GCS) of 13. Initial vital signs revealed tachycardia (123 beats per minute, regular and strong pulse), an axillary temperature of 38.0°C, and others were within normal limits. Physical examination revealed icteric sclera, hepatosplenomegaly, and epigastric tenderness. Other neurological examinations were normal.

Laboratory tests revealed parasitemia with a density of 196,655 parasites/μL, mild anemia, thrombocytopenia, and moderate hyponatremia. Impaired liver and kidney function were observed (table 1). “Coca-cola”-colored urine was noted following urinary catheter insertion. The urine output was 0.3 mL/kg/hour for the last 6 hours of observation, therefore this patient was diagnosed with AKI.

**Table 1:** Laboratory values from admission day until follow up

Parameters (normal values)	Day 1	Day 2	Day 3	Day 9
**Blood test**				
**HB** g/dL (12.8-16.8)	10.9	8.3	7.5	7.7
**WBC** 10^3^/μL (5.0-10.0)	6.06	7.49	7.36	8.74
**PLT** 10^3^/μL (150-450)	27	48	72	192
**Blood smear** parasite/μL	196,665	1,673	Negative	Negative
**Tot. bil.** mg/dL (0.3-1.0)	4.72	1.1	0.54	0.8
**Dir. bil.** mg/dL (<0.2)	3.05	0.8	0.38	0.33
**AST** U/dL (<38)	194	86	66	32
**ALT** U/dL (<41)	39.1	30	57	57
**Urea** mg/dL (10.0-50.0)	90.4	101	44.4	18.3
**Creatinine** mg/dL (<1.2)	0.8	0.7	0.6	0.5
**eGFR** mL/min/1.73m^2^ (≥90)	76.6	87.5	102.1	122.5
**Sodium** mmol/L (136-145)	128	133	134	-
**Potassium** mmol/L (3.5-5.1)	4.9	3.6	3.2	-
**Urinary test**				
**Color**	Black	Tea colored	Yellow	Light yellow
**RBC** /HPF (≤1)	>400	-	-	0-1
**WBC** /HPF(≤5)	5-10	-	-	1-4
**Protein** (negative)	2+	-	-	Negative
**Bilirubin** (negative)	1+	-	-	Negative
**Nitrite** (negative)	+	-	-	Negative
**Bacteria** (negative)	+	-	-	Negative

Hb; hemoglobin. WBC; white blood cell. PLT; platelet. Tot.bil.; serum total bilirubin. Dir.bil; serum direct bilirubin. AST; aspartate aminotransferase. ALT; alanine aminotransferase. eGFR; estimated glomerular filtration rate. RBC; red blood cell.

The patient was hospitalized with BWF as the primary diagnosis based on the signs and symptoms of fever, “coca-cola”-colored urine, anemia, jaundice, and AKI. Because parenteral antimalarials were unavailable at that time, this patient was treated with oral antimalarials, DHP for two more days, with close monitoring of vital signs and urine output, as well as intravenous antibacterial and other supportive medical care, such as fluid replacement and electrolyte management.

On the second day of care, there was a significant improvement and the patient only complained of mild epigastric pain after completing the second dose of DHP. Physical examination revealed no icteric sclera, a GCS of 15, and improved urine color. Blood tests revealed a decrease in parasite density, a decrease in hemoglobin, an increase in urea, and improvements in other parameters (table 1).

Recovery was favorable on the third day of care. The patient did not experience any symptoms, urine color was normalized, and laboratory parameters were improved (table 1). The patient was discharged from the hospital on this day. Six days after being discharged, the patient returned for a medical check-up without any complaints and demonstrated significant improvement in laboratory results (table 1).

## Discussion

Malaria is a vector-borne disease commonly found in some endemic areas of Indonesia, with Papua having the highest prevalence (Health Ministry of Republic of Indonesia 2018). Historically, BWF was thought to be a rare form of severe malaria. However, Mohapatra et al. (2020) and Olupot et al. (2017) concluded that BWF is no longer a rare severe malaria form. A study in India conducted by Mohapatra et al. (2020) discovered ten patients with BWF among 100 malaria patients. Olupot et al. found that 147 of 1,085 children with positive malaria on peripheral blood smear examination were diagnosed as BWF.

BWF can occur in both children and adults, as well as in all types of malaria, with *Plasmodium falciparum* infection having the highest prevalence (Lon et al., 2014; Mahamadou et al., 2019; Mohapatra et al., 2020; Olupot et al., 2017). The syndrome of BWF is frequently diagnosed only after malaria patients are noted to have dark-colored urine, as was discovered in this case (Lon et al., 2014).

The exact etiology of BWF remains unclear. Apart from being associated with *Plasmodium falciparum* infection, most studies and case reports have linked it to amino-alcohol antimalarials, particularly quinine (Bodi et al., 2013; Mahamadou et al., 2019; Mohapatra et al., 2020; Oluput et al., 2017). Tran et al. (1996) reported that G6PD deficiency was found in 54% of BWF cases. Amino-alcohol antimalarials and G6PD deficiency were thought to share a similar mechanism, which both produce oxidative stress leading to massive intravascular hemolysis (Huggan et al., 2018). The other risk factors are the use of artemisinin derivatives and recurrent malaria infections (Lon et al., 2014). In this case, the patient had a proven *Plasmodium falciparum* infection, which was the major risk factor for BWF. Recurrent malaria infection and the use of artemisinin derivatives as antimalarials were also risk factors in this case. Although this was the recurrent malaria infection in this patient, the syndrome of BWF developed several hours following the first dose of DHP, leading us to believe that artemisinin derivatives were the cause of BWF in this patient. Lon et al. (2014) found a similar result, that BWF developed in their patient after completing the first two doses of DHP. Nevertheless, further research is needed to prove it.

The mortality rate of BWF ranges from 2 to 20% (Mohapatra et al., 2020; Olupot et al., 2017; Tran et al., 1996). Despite that artemisinin derivatives are considered to be one of the risk factors of BWF, the World Health Organization (2021) recommends artemisinin derivatives (artesunate or artemether) as the first line of treatment for severe malaria. Mahamadou et al. (2019) also described successful intramuscular artemether treatment of BWF. The studies conducted by Barber et al. (2016) and Mohapatra et al. (2020) showed a high efficacy of intravenous artesunate in treating BWF. To the best of our knowledge, there is no published article that describes the successful treatment of BWF with oral antimalarials. In this case, we treated our patient with oral antimalarials, because parenteral antimalarials were unavailable at that time. Although DHP appeared to be the triggering factor of BWF in this case, we had no choice but to complete a 3-day course of DHP under close monitoring for any signs of clinical deterioration. Surprisingly, remarkable clinical and laboratory improvements were seen following the second and third doses of DHP along with adequate supportive medical care.

Limitations of this study were due to limited resources, such as some tests were not available in our context, including detection of G6PD deficiency and hemoglobin in urine. Blood culture and other infection markers were also not done to exclude concomitant bacteremia.

## Conclusion

BWF is one of the severe forms of malaria that is recommended to be treated with a parenteral antimalarial. The unavailability of parenteral antimalarials makes oral antimalarials a possible alternative treatment for BWF. In addition, close monitoring and supportive medical care are critical in the treatment of BWF. More research is clearly needed to learn more about the association between artemisinin derivatives and the occurrence of BWF, as the onset of BWF in this case occurred after the first dose of DHP.

## Declaration of Competing Interest

The authors declare no competing interests.
